# Association of California Immigrants' Avoidance of Public Programs Due to Immigration Concerns With Delayed Access to Health Care

**DOI:** 10.1001/jamanetworkopen.2022.46525

**Published:** 2022-12-13

**Authors:** Joelle Wolstein, Susan H. Babey, Sean Tan, Riti Shimkhada, Ninez A. Ponce

**Affiliations:** 1 UCLA Center for Health Policy Research, UCLA Fielding School of Public Health, Los Angeles, California; 2 Department of Health Policy and Management, UCLA Fielding School of Public Health, Los Angeles, California

## Abstract

This cross-sectional study examines the association of avoidance of public programs among California immigrants with delayed access to health care services and prescriptions owing to concerns about how their interaction with these services may affect their immigration status.

## Introduction

Since 1882, the US has had exclusion rules to prevent permanent residency applicants from entering and remaining in the country if they are deemed a public charge or likely to become primarily dependent on the government to meet basic needs.^1^ In 2018, the Trump Administration proposed additional public programs be considered in determining persons likely to be deemed a public charge, including Medicaid, Supplemental Nutrition Assistance Program (SNAP), and some housing assistance programs. Changes were implemented in 2019 but eventually revoked.

The Trump Administration’s changes to public charge rules contributed to avoidance of public programs among immigrants, even program-eligible immigrants, because immigrants believed access would affect their immigration status or that of a family member.^2^ Decreased participation in Medicaid and SNAP can negatively impact health and access to health care. This study examines the association of avoidance of public programs due to immigration concerns with access of health care services and prescription medication in California, home to the largest immigrant population in the US.

## Methods

In this cross-sectional study, we used data from the 2019 California Health Interview Survey^3^ to examine the association of avoidance of public programs due to immigration concerns with delays in obtaining medical care and prescriptions among low-income (<200% Federal Poverty Level) immigrant adults (eMethods in the Supplement). Race and ethnicity were self-reported as Latino, non-Latino Asian, non-Latino White, and other (including non-Latino American Indian and Alaska Native, Black or African American, and Native Hawaiian and Pacific Islander, self-described other, and more than 1 race) and included to account for different immigrant experiences across groups. Estimates were weighted to be representative of the California population and adjusted for complex survey design effects. Logistic regression models computed adjusted odds ratios (ORs) and 95% CIs to test associations. The University of California, Los Angeles, Institutional Review Board approved this research. Written informed consent was obtained from web-based respondents and obtained orally from telephone-based participants. This study followed the STROBE reporting guidelines.

## Results

Among low-income immigrants in California, 46.1% were men and 53.9% were women; 69.2% were Latino and 23.2% were non-Latino Asian; and 22.9% were uninsured for all or part of the past year (Table 1). Avoidance of public programs in the past year for fear of harming their immigration status was reported by 13.7%. Logistic regression analyses adjusting for all variables (Table 2) revealed that, compared with low-income immigrants who did not avoid public programs, those who did had more than twice the odds of delaying needed medical care (OR, 2.39 [95% CI, 1.39-4.12]) and filling prescriptions (OR, 2.36 [95% CI, 1.21-4.62]).

**Table 1.** Characteristics of California Immigrant Adults With Incomes Less Than 200% FPL^a^

Characteristic	No. (weighted %) [SE]^b^
Age, y	
18-34	213 (27.4) [1.7]
35-49	342 (29.9) [1.5]
50-64	426 (26.2) [1.4]
≥65	413 (16.6) [1.2]
Gender^c^	
Male	569 (46.1) [1.8]
Female	825 (53.9) [1.8]
Race and ethnicity	
Asian	398 (23.2) [1.0]
Latino	759 (69.2) [1.1]
White	191 (5.4) [0.6]
Other^d^	46 (2.2) [0.6]
Income	
0%-99% FPL	555 (44.2) [1.9]
100%-199% FPL	839 (55.8) [1.9]
Educational attainment	
<High school	303 (44) [1.5]
High school graduate	674 (40.7) [1.4]
College graduate	417 (15.3) [1.1]
Family composition	
Single no children	529 (29.8) [1.4]
Single with children	174 (18.7) [1.7]
Married no children	384 (23.1) [1.4]
Married with children	310 (28.3) [1.8]
Urbanicity	
Urban	1250 (92.8) [0.9]
Rural	144 (7.2) [0.9]
Insurance coverage	
Uninsured all or part of year	239 (22.9) [1.5]
Insured all year	1155 (77.07) [1.5]
Avoided public programs in past year	
Avoided	142 (13.7) [1.3]
Did not avoid	1252 (86.3) [1.3]
Delayed needed medical care in past year	
Delayed	217 (14.9) [1.4]
Did not delay	1177 (85.1) [1.4]
Delayed filling prescription medication in past year	
Delayed	166 (11.34) [1.1]
Did not delay	1288 (88.66) [1.1]

Abbreviation: FPL, Federal Poverty Level.

^a^ Data are from the 2019 California Health Interview Survey and include 1394 respondents.

^b^ Estimates are weighted to be representative of the California population and are adjusted for complex survey design effects.

^c^ Respondents were asked how they describe themselves.

^d^ Includes non-Latino American Indian or Alaska Native, non-Latino Black or African American, non-Latino Native Hawaiian or other Pacific Islander, respondents describing their race or ethnicity as “other,” and respondents selecting 2 or more races or ethnicities.

**Table 2.** Logistic Regression of Factors Associated With Delays in Medical Care and Delays in Filling Medication Prescriptions Among Low-Income California Immigrant Adults^a^

Variable	Delayed medical care		Delayed prescription filling	
	Adjusted OR (95% CI)^b^	*P* value	Adjusted OR (95% CI)^b^	*P* value
Avoided public programs in past year				
Avoided	2.39 (1.39-4.12)	.002	2.36 (1.21-4.62)	.01
Did not avoid	1 [Reference]	NA	1 [Reference]	NA
Age	0.99 (0.98-1.01)	.33	0.99 (0.96-1.01)	.20
Gender^c^				
Female	1.13 (0.76-1.68)	.54	1.26 (0.83-1.94)	.28
Male	1 [Reference]	NA	1 [Reference]	NA
Race and ethnicity				
Asian	1.09 (0.54-2.19)	.81	0.93 (0.42-2.09)	.86
Latino	1.03 (0.55-1.92)	.92	1.67 (0.8-3.5)	.17
White	1 [Reference]	NA	1 [Reference]	NA
Other^d^	2.64 (0.58-11.92)	.21	5 (0.67-37.52)	.12
Income, FPL				
0%-99%	0.8 (0.51-1.23)	.30	0.67 (0.38-1.19)	.17
100%-199%	1 [Reference]	NA	1 [Reference]	NA
Educational attainment				
Less than high school	0.84 (0.41-1.71)	.62	1.62 (0.63-4.15)	.31
High school graduate	0.92 (0.53-1.6)	.77	2.05 (1.12-3.78)	.02
College graduate	1 [Reference]	NA	1 [Reference]	NA
Family composition				
Single with children	0.95 (0.46-1.94)	.89	0.26 (0.12-0.59)	.002
Single no children	0.57 (0.29-1.14)	.11	0.76 (0.37-1.54)	.44
Married with children	0.76 (0.39-1.51)	.43	0.6 (0.3-1.19)	.14
Married with no children	1 [Reference]	NA	1 [Reference]	NA
Urbanicity				
Rural (vs urban)	0.45 (0.22-0.9)	.03	1.14 (0.43-3.01)	.80
Urban	1 [Reference]	NA	1 [Reference]	NA
Insurance coverage				
Uninsured all or part of year	1.59 (1.01-2.51)	.05	0.87 (0.52-1.47)	.61
Insured all year	1 [Reference]	NA	1 [Reference]	NA

Abbreviations: FPL, Federal Poverty Level; NA, not applicable; OR, odds ratio.

^a^ Data are from the 2019 California Health Interview Survey and include 1394 respondents.

^b^ Estimates are weighted to be representative of the California population and are adjusted to account for complex survey design effects.

^c^ Respondents were asked how they describe themselves.

^d^ Includes non-Latino American Indian or Alaska Native, non-Latino Black or African American, non-Latino Native Hawaiian or other Pacific Islander, respondents describing their race or ethnicity as “other,” and respondents selecting 2 or more races or ethnicities.

## Discussion

Delays in accessing needed health care can have negative health consequences, including increased risk of mortality.^4^ We found that low-income California immigrants who avoided public programs owing to fear of harming their immigration status were twice as likely to delay needed medical care or prescription fills.

Our findings are consistent with other studies and add to the growing evidence of how exclusionary immigration policies affect public program participation and harm health. Other research^5^ showed that 16% of immigrant adults avoided government benefit programs such as Medicaid or SNAP in 2019 owing to proposed public charge changes. Another study found that the rule change led to declines in children’s enrollment in Medicaid and the Women, Infants and Children assistance program.^6^

Although our study is from a state with a large immigrant population, the experience of California immigrants may not generalize to other immigrants around the country. Additionally, respondents were asked if they had decided not to apply for public programs, which may not capture respondents who were enrollees but decided to discontinue participation. Other limitations include the cross-sectional nature of the survey and how beliefs about public charge may have fluctuated during the year, depending on media coverage or other factors not measured.

US immigration policy over time and place varies in the level of institutional exclusion of immigrants,^1^ and the Biden Administration’s revocation of the 2018 public charge rule may mitigate the adverse findings we report. Thus, any future formulation of exclusionary immigration policies must consider the negative effects on health access and health on the immigrant population in the US.

## Notes

Supplement 1. **eMethods.** Data Sources, Measures, and Analysis

Supplement 2.
Data Sharing Statement
