# Supplementary material for: Association of California Immigrants' Avoidance of Public Programs Due to Immigration Concerns With Delayed Access to Health Care
Source: JAMA Netw Open. 2022 Dec 13;5(12):e2246525. doi: 10.1001/jamanetworkopen.2022.46525 (PMC9856315; doi:10.1001/jamanetworkopen.2022.46525)
Supplement: Supplement 1. — eMethods. Data Sources, Measures, and Analysis [file jamanetwopen-e2246525-s001.pdf]

# Supplementary Online Content

Wolstein J, Babey SH, Tan S, Shimkhada R, Ponce NA. Association of California immigrants' avoidance of public programs due to immigration concerns with delayed access to health care. *JAMA Netw Open*. 2022;5(12):e2246525. doi:10.1001/jamanetworkopen.2022.46525

**eMethods.** Data Sources, Measures, and Analysis

This supplementary material has been provided by the authors to give readers additional information about their work.

© 2022 Wolstein J et al. *JAMA Network Open*.

**eMethods.** Data Sources, Measures, and Analysis

*Data source and population*

Data for this study were from the adult sample of the 2019 California Health Interview Survey (CHIS). CHIS is the largest state health survey with a module on immigration status. It is designed to be representative of California's non-institutionalized population. In 2019, CHIS data were collected via an address-based sample methodology with multimode data collection that took place on the web or by telephone. One randomly selected adult (aged 18 or older) was interviewed in each household. Interviews were available in English, Spanish, Chinese (Cantonese and Mandarin dialects), Vietnamese, Korean, and Tagalog. The adult response rate after household screening was 72.3%. Detailed descriptions of CHIS methodology are available elsewhere.[4] A total of 22,160 adults completed the survey in 2019. Analyses were limited to adult California immigrants (those born outside the U.S.). Analyses were also limited to those with household incomes below 200% Federal Poverty Level (FPL) (n=1,394) because those with higher incomes are much less likely to be eligible for public programs such as Medi-Cal and CalFresh (California's SNAP program).

*Measures*

Adult responses to two questions were used to assess the outcomes, whether individuals delayed prescription medications or needed medical care: (1) "During the past 12 months, did you delay or not get a medicine that a doctor prescribed for you?" and (2) "During

© 2022 Wolstein J et al. *JAMA Network Open*.

the past 12 months, did you delay or not get any other medical care you felt you needed—such as seeing a doctor, a specialist, or other health professional?" Responses were categorical.

*Independent variables of interest*

Adult respondents born outside the U.S. or its territories were asked, "Was there ever a time when you decided not to apply for one or more noncash government benefits, such as Medi-Cal, food stamps, or housing subsidies, because you were worried it would disqualify you or a family member from obtaining a green card or becoming a U.S. citizen?" Those who responded "yes" were also asked whether this had happened in the past 12 months. Responses to these questions were used to construct a dichotomous indicator: avoided programs in the past year vs. did not avoid programs in past year or never avoided programs. This study focused on avoiding programs in the past 12 months because both outcome measures refer to accessing care in the past year.

*Covariates*

The following sociodemographic characteristics were included as covariates in all models: age, gender, race/ethnicity, household income, education, family composition, urbanicity, and insurance coverage. Age was included as a continuous variable. Race/ethnicity was self-reported and categorized as Latino, Non-Latino Asian, Non-Latino White, and other race/ethnicity. Due to small sample sizes among our analytic sample of low-income immigrants born outside the U.S., we created an "other" race/ethnicity category that included Non-Latino American Indian/Alaska Native, Non-Latino Black or African American, Non-Latino Native

© 2022 Wolstein J et al. *JAMA Network Open*.

Hawaiian/Pacific Islander, respondents describing their race as “other”, and respondents selecting two or more races.  Household income was reported as a percentage of the federal poverty level: 0-99% versus 100-199%. Education was categorized as less than high school, high school graduate, and college graduate or higher. Family composition was categorized as single with no children, single with children, married with no children, and married with children. Households were assigned to urbanicity levels (urban vs rural) based on population density of the household’s zip code and surrounding areas. Health insurance coverage was dichotomized as uninsured for all or part of the year and insured all year.

*Statistical Analyses*

Multinomial logistic regression models were run to estimate the association of avoidance of public programs with delays in medical care (model 1) and delays in filling prescription medication (model 2). Data were analyzed using SAS. Analyses were weighted to be representative of the California population and adjusted for the complex survey design of the California Health Interview Survey.

© 2022 Wolstein J et al. *JAMA Network Open*.
