# Supplementary material for: Association of California Immigrants' Avoidance of Public Programs Due to Immigration Concerns With Delayed Access to Health Care
Source: JAMA Netw Open. 2022 Dec 13;5(12):e2246525. doi: 10.1001/jamanetworkopen.2022.46525 (PMC9856315; doi:10.1001/jamanetworkopen.2022.46525)
Supplement: Supplement 2. — Data Sharing Statement [file jamanetwopen-e2246525-s002.pdf]

Data Sharing Statement

Wolstein. Association of California Immigrants' Avoidance of Public Programs Due to Immigration Concerns With Delayed Access to Health Care. *JAMA Netw Open*. Published December 13, 2022. doi:10.1001/jamanetworkopen.2022.46525

Data
Data available: No

Additional Information
Explanation for why data not available: This study used restricted data from the California Health Interview Survey. These data are not publically available. Researchers can access the data through the UCLA Center for Health Policy Research Data Access Center.
